# Ferric Ammonium Citrate Upregulates PD-L1 Expression through Generation of Reactive Oxygen Species

**DOI:** 10.1155/2022/6284124

**Published:** 2022-01-17

**Authors:** Eun Jung Choi, Chang Hyun Jeon, In-Kyu Lee

**Affiliations:** ^1^Research Institute of Aging and Metabolism, Kyungpook National University, Daegu 41404, Republic of Korea; ^2^Department of Biomedical Science, School of Medicine, Kyungpook National University, Daegu 41566, Republic of Korea; ^3^Department of Internal Medicine, School of Medicine, Kyungpook National University, Kyungpook National University Hospital, Daegu 41944, Republic of Korea

## Abstract

Iron plays an important role in macrophage polarization by altering metabolic and redox status. However, the impact of iron on the immune status of macrophages is still controversial. In this study, we report that ferric ammonium citrate (FAC) upregulates PD-L1 expression in macrophages. FAC not only altered the phenotype of macrophages but also led to enriching immune-modulatory T cell subsets. Since iron is known to be a constituent of coenzymes facilitating metabolic processes in mitochondria, we examined the metabolic status of FAC-overloaded macrophages by measuring the oxygen consumption rate (OCR) and the represented coenzyme, aconitase. In addition to enhancement of metabolic processes, FAC accelerated the Fenton reaction in macrophages, which also contributed to the facilitation of oxygen consumption. We reasoned that the enhancement of the OCR leads to the production of reactive oxygen species (ROS), which are directly linked to PD-L1 induction. Using ferrostatin, rotenone, and N-acetyl-L-cysteine, we confirmed that metabolic and redox regulation is responsible for FAC-mediated PD-L1 expression. Furthermore, we suggested that FAC-induced ROS production may explain FAC-mediated pro- and anti-inflammatory responses in macrophages. These findings may extend our understanding of regulating iron concentration during immune checkpoint therapy in cancer patients.

## 1. Introduction

Macrophages play key roles in regulating the innate and adaptive immune systems in response to environmental milieu by polarizing toward either M1 (classically activated) or M2 (alternatively activated) macrophages [1]. Metabolic reprogramming in macrophages is an important factor in determining such polarization status [2–5]. Upon exposure to a trigger, a naïve macrophage polarizes into M1 by turning on proinflammatory processes, including the expression of costimulatory molecules and the production of cytokines. Metabolically, M1 shifts glucose usage from oxidative phosphorylation (OXPHOS) in mitochondria to glycolysis, which supports the abrupt demand for energy during activation [4, 5]. The reduced flux in turn generates two metabolic breaks in mitochondria, which leads to the accumulation of citrate and succinate [4, 6]. Inhibition of aconitase activity during M1 is an important factor that hinders the flux in the tricarboxylic acid (TCA) cycle [7, 8]. The accumulation of citrate facilitates the lipid membrane expansion that is essential for the transportation and secretion of cytokines; moreover, the excess succinate aggravates hypoxia-induced factor 1*α*- (HIF1*α*-) mediated inflammation, which helps sustain M1 status [6, 9]. On the other hand, M2 macrophages predominantly produce energy via OXPHOS by facilitating TCA flux in mitochondria [10]. The expression of PD-L1, one of the costimulatory molecules that promotes polarization toward M2, requires OXPHOS-dominant metabolic reprogramming [11–13].

Iron governs various metabolic processes within macrophages that are directly correlated with their immunologic function [14–16]. Iron is a well-known component of the coenzymes in the TCA cycle or electron transport chain (ETC), and the correlation between these factors and iron-mediated macrophage polarization has been investigated [17]. Iron comprises several coenzymes in mitochondria to support the energy production processes. For example, iron forms the [4Fe-4S] cluster in aconitase, an enzyme that interconverts citrate and isocitrate in the TCA cycle, whose activity is crucial for citrate oxidation [7, 8]. Moreover, iron forms [2Fe-2S] or [4Fe-4S] in complex I, which constitutes iron-sulfur clusters that are responsible for electron delivery. Facilitation of the OXPHOS-dependent pathway successively enhances the flow of electrons in the ETC, which promotes reactive oxygen species (ROS) generation during the process [18–20]. Iron further accelerates ROS generation by the Fenton reaction, which converts hydrogen peroxide into hydroxyl free radicals [21]. Several papers report the impact of iron on the immune function of macrophages, although conflicting results have been reported [14, 22–25]. Therefore, this study was conducted with an aim to elucidate the seemingly conflicting results of iron-mediated inflammatory responses in macrophages by using FAC as an iron source. Moreover, we investigated whether FAC increases PD-L1 expression in macrophages to induce M2 polarization and delineated the effects of FAC on CD4^+^ T cell differentiation. Next, we investigated the metabolic effect of FAC in mitochondria, and by using various ROS inhibitors, we ascertained whether the contradictory immunologic phenomena stem from iron-mediated ROS production.

## 2. Materials and Methods

### 2.1. Materials

Ferrostatin-1, rotenone, and N-acetyl-L-cysteine (NAC) were all purchased from Sigma (St. Louis, MO). The ELISA kit for the measurement of IL-1*β* was purchased from eBioscience (San Diego, CA), and aconitase activity was measured by an aconitase activity colorimetric assay kit (BioVision Inc., San Francisco, CA).

### 2.2. Animals

Eight-week-old 20 g male C57BL/6J and BALB/c mice were purchased from DooYeol Biotech (Seoul, South Korea). All animal studies were performed according to protocols approved by Kyungpook National University (Permit Number: 2019-0003) and under recommendations for the proper use and care of the specific pathogen-free housing facility at Kyungpook University. The removal of bone marrow was performed under isoflurane anesthesia.

### 2.3. Isolation of Bone Marrow-Derived Macrophages

Bone marrow-derived macrophages (BMDMs) were generated from the bone marrow of C57BL/6J mice via incubation at 37°C with 5% CO_2_ in DMEM (WelGENE, South Korea) supplemented with 10% fetal bovine serum (FBS), 1% penicillin/streptomycin, and 20 ng/mL macrophage colony-stimulating factor (M-CSF) (PeproTech, Rocky Hill, NJ). The cells were harvested at day 5 of expansion and were used for subsequent assays.

### 2.4. Flow Cytometry

Cells were stained with PE-Cy7 anti-F4/80, PE-Cy7 anti-CD11c, PerCP anti-CD3, PE anti-PD-L1, APC anti-CD86, FITC-CD206, PE anti-Foxp3, APC anti-CD25, and APC anti-IFN*γ* (BioLegend, San Diego, CA). Labile cell iron was measured using 10 *μ*M calcein acetoxymethyl (calcein_AM, Thermo Fisher Scientific, MA). Lipid peroxidation was assessed by C11-BODIPY 581/591 (Thermo Fisher Scientific, MA), and the amount of ROS was measured by a DCFDA cellular ROS detection assay kit (Abcam, Cambridge, UK). CD4^+^IFN*γ*^+^ cells were detected by an intracellular staining kit (Fixation/Permeabilization Solution Kit, Franklin Lakes, NJ), and CD4^+^CD25^+^Foxp3^+^ cells were measured using a Foxp3/transcription factor staining buffer set (eBioscience). All data were analyzed using FACS LSR Fortessa cytometry with BD CELL Quest Pro software.

### 2.5. Mixed Lymphocyte Reaction

CD4^+^ T cells were isolated from BALB/c splenocytes using a CD4^+^ T cell isolation kit (Miltenyi Biotec, Bergisch Gladbach, Germany). Next, 5 × 10^5^ CD4^+^ T cells (from BALB/c) and 5 × 10^5^ cells of macrophages (from C57BL/6J) were cocultured for 5 days with 2 mM FAC or vehicle control (water). CD4^+^IFN*γ*^+^ cells were subjected to Cell Stimulation Cocktail (plus protein transport inhibitors, eBioscience) for 5 h, and percentages of CD4^+^IFN*γ*^+^cells were detected using flow cytometry.

### 2.6. Measurement of the Oxygen Consumption Rate

The oxygen consumption rate (OCR) was measured using a Seahorse XF-96 Flux Analyzer (Seahorse Biosciences, Billerica, MA). The culture medium consisted of DMEM supplemented with 10% FBS and penicillin-streptomycin-amphotericin B. BMDMs were seeded in an XF-96 culture plate at a density of 1 × 10^5^ cells/well and incubated overnight. The cells were treated with FAC for 16 h. The assay medium comprised the XF base medium (Seahorse Biosciences) supplemented with 5.5 mM D-glucose (Sigma-Aldrich), 1 mM sodium pyruvate (Sigma-Aldrich), and 1X GlutaMAX™ (Gibco), adjusted to pH 7.4. The inhibitors were used in the following concentrations: oligomycin A (2 *μ*mol/L; Sigma-Aldrich), **c**arbonyl cyanide-4 (trifluoromethoxy) phenylhydrazone (FCCP, 1 *μ*mol/L; Sigma-Aldrich), rotenone (3 *μ*mol/L; Sigma-Aldrich), and antimycin A (3 *μ*mol/L; Sigma-Aldrich). In short, oligomycin inhibits ATP synthase (complex V), and the decrease in OCR represents cellular ATP production. FCCP is an uncoupling agent to calculate spare respiratory capacity. A combination of rotenone, a complex I inhibitor, and antimycin A, a complex III inhibitor, shuts down mitochondrial respiration and enables the calculation of nonmitochondrial respiration.

### 2.7. Statistical Analysis

All data are presented as means ± SD of three to four independent experiments. Individual data points were compared by Student's *t*-test. The analysis was performed using SPSS software (version 22.0). Differences between groups were considered significant at *P* < 0.05.

## 3. Results

### 3.1. FAC-Treated Macrophages Upregulate PD-L1 Expression

**Figure 1:**
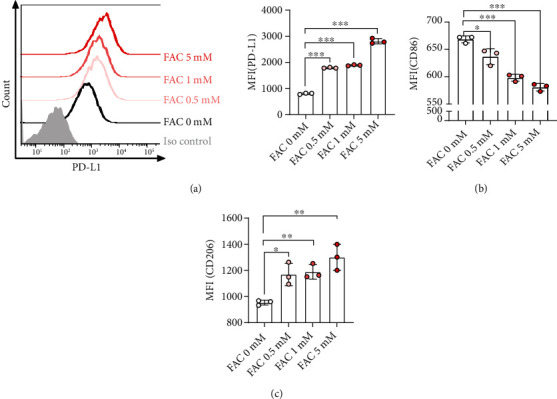
Effects of FAC on macrophage PD-L1 expression. BMDMs were incubated with FAC at concentrations of 0.5, 1, and 5 mM for 16 h. PD-L1 (a), CD86 (b), and CD206 (c) in F4/80^+^ macrophages were measured by flow cytometry. The scatterplot with the bar graph illustrates the mean fluorescence intensity (MFI) of each surface marker. ∗ indicates significant differences (^∗^*P* < 0.05, ^∗∗^*P* < 0.01, and ^∗∗∗^*P* < 0.001) from the control group.

To confirm the role of FAC in macrophage polarization, we first investigated the change in the expression of costimulatory molecules in macrophages. Interestingly, FAC upregulated PD-L1 expression in a dose-dependent manner (Figure 1(a)). Meanwhile, CD86 expression in macrophages was decreased and CD206 was upregulated, which represent M1 and M2 polarization markers, respectively (Figures 1(b) and 1(c)). In the absence of other triggers, FAC alone induced features of M2 in macrophages.

### 3.2. FAC-Treated Macrophages Functionally Regulate Th1 Cells and Treg Cells

**Figure 2:**
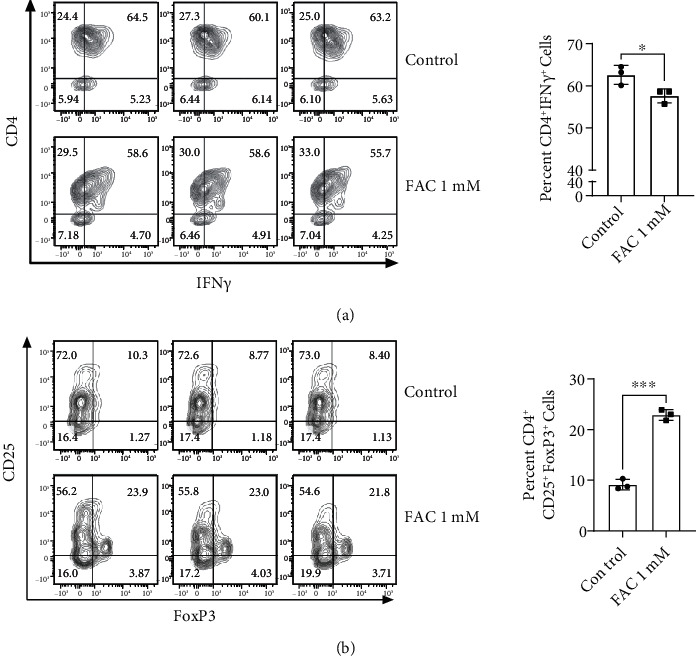
Effects of FAC-treated macrophages on CD4^+^ T cell polarization. C57BL/6J-derived macrophages were treated with 1 mM FAC for 16 h. After washing with media, cells were coincubated with BALB/c-originated CD4^+^ T cells for 5 d. The percentages of CD4^+^IFN*γ*^+^ Th1 cells (a) and CD4^+^CD25^+^Foxp3^+^ Treg cells (b) were measured by flow cytometry. ∗ indicates significant differences (^∗^*P* < 0.05, ^∗∗^*P* < 0.01, and ^∗∗∗^*P* < 0.001) from the control group.

As antigen-presenting cells, polarized macrophages confer the immunologic phenotype of T cells. M1 macrophages drive the Th1 response, whereas M2 conditions cause naïve T cells to differentiate into anti-inflammatory Treg cells [26]. To delineate how FAC-treated macrophages interact with T cells, mixed lymphocyte reaction (MLR) was performed [27]. After incubation of FAC for 16 h, macrophages were cocultured with splenic CD4^+^ T cells isolated from BALB/c. As shown in Figures 2(a) and 2(b), FAC-treated macrophages markedly reduced the percentage of CD4^+^IFN*γ*^+^ Th1 cells and increased that of CD4^+^CD25^+^Foxp3^+^ Treg cells. Thus, FAC not only induced M2 polarization but also had a significant impact on the induction of Treg cells.

### 3.3. FAC Enhances Mitochondrial Function and Lipid Peroxidation

**Figure 3:**
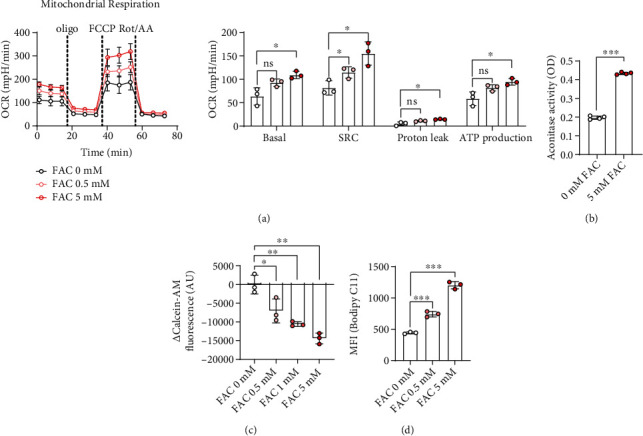
Effects of FAC on mitochondrial function. 1 × 10^6^ cells/mL BMDMs were stimulated with indicated concentrations of FAC for 16 h. (a) The OCR was measured using a Seahorse XF-96 Flux Analyzer. The scatterplot with the bar graph illustrates calculated respiratory parameters. (b) Aconitase activity was measured by ELISA, and (c) the amount of intracellular labile iron was given as the delta (Δ) of the fluorescence to initial fluorescence. (d) The amount of lipid peroxidation was estimated by the mean fluorescence intensity (MFI) of C11-BODIPY using flow cytometry. ∗ indicates significant differences (^∗^*P* < 0.05, ^∗∗^*P* < 0.01, and ^∗∗∗^*P* < 0.001) from the control group.

As iron is a component of various coenzymes that facilitates the mitochondrial metabolic processes, we determined the metabolic role of FAC in macrophage polarization [14–16]. To verify the effect of FAC on mitochondrial respiration, the OCR was assessed. FAC significantly facilitated not only the basal level of oxygen consumption but also the mitochondrial-dependent ATP production during macrophage polarization (Figure 3(a)). FAC has been reported to increase aconitase enzymatic activity, as demonstrated in prostatic carcinoma cells [7, 8]. We confirmed that 5 mM FAC enhances aconitase activity in macrophages, which accelerates citrate oxidation to enhance oxygen consumption (Figure 3(b)). This finding is consistent with recent reports that PD-L1 expression can be promoted by OXPHOS-dependent mitochondrial energy utilization [11, 12].

Redox-active ferrous ions (Fe^2+^) not only alter the metabolic status of macrophages but also result in increased oxygen consumption [24]. Iron is present in the form of inert Fe^3+^ in the extracellular space and converted into redox-active Fe^2+^ ions [16, 28]. Intracellular Fe^2+^ produces hydroxyl radicals, termed the Fenton reaction, which results in lipid peroxidation [21]. Thus, we examined whether FAC (Fe^3+^) generates intracellular redox-active Fe^2+^ and the resultant lipid peroxidation status. FAC enhanced redox-active Fe^2+^ in macrophages, and thereby, lipid peroxidation was significantly increased in a dose-dependent manner (Figures 3(c) and 3(d)). Collectively, we found that FAC enhances OXPHOS-dependent metabolic processes through citrate oxidation and the induction of Fenton reaction, which successively results in the accumulation of peroxidized lipids.

### 3.4. FAC-Induced ROS Production Mediates Both Inflammasome Activation and PD-L1 Upregulation

**Figure 4:**
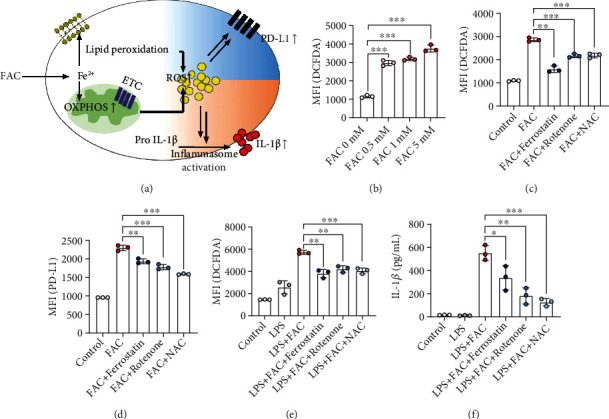
Role of ROS in FAC-mediated IL-1*β* and PD-L1 production. (a) Schematic diagram explaining FAC-mediated inflammatory response in macrophages. (b) BMDMs were incubated with FAC at concentrations of 0.5, 1, and 5 mM for 16 h. ROS production in F4/80^+^ macrophages was measured by flow cytometry. (c, d) BMDMs were incubated with FAC at a concentration of 0.5 mM FAC in the presence of 2 *μ*M ferrostatin, 0.1 mM rotenone, and 0.2 *μ*M NAC for 16 h. ROS (c) and PD-L1 production (d) in F4/80^+^ macrophages were measured by flow cytometry. (e, f) BMDMs were pretreated with LPS 500 ng/mL for 3 h. After priming, 0.5 mM FAC, 2 *μ*M ferrostatin, 0.1 mM rotenone, and 0.2 *μ*M NAC were coincubated with 1 mM ATP for 4 h. ROS (e) and IL-1*β* production (f) were estimated.

To this point, we investigated the role of FAC in facilitating the oxygen consumption processes during M2 polarization. FAC enhances the production of IL-1*β*, one of the proinflammatory cytokines; therefore, we investigated the mechanisms through which FAC simultaneously manifests both the pro- and anti-inflammatory features [24]. The FAC-mediated enhancement of OXPHOS and the Fenton reaction successively lead to ROS accumulation. We reasoned that FAC-mediated metabolic and redox status induces ROS production, and it may lead to enhanced PD-L1 expression and inflammasome activation to produce IL-1*β* at the same time (Figure 4(a)). As expected, FAC increased ROS production in a dose-dependent manner (Figure 4(b)). We examined whether ROS produced by FAC-mediated OXPHOS and the Fenton reaction is responsible for IL-1*β* and PD-L1 expression. Incubation of FAC with ferrostatin (a lipid peroxidation process inhibitor), rotenone (a complex I inhibitor), and NAC (an ROS inhibitor) for 16 h significantly reduced FAC-induced ROS, which abrogated FAC-induced PD-L1 expression in macrophages (Figures 4(c) and 4(d)). IL-1*β* production involves priming and inflammasome activation processes [29]. Once pro-IL-1*β* is expressed, inflammasomes are triggered by various stimuli, including ATP, nigericin, and ROS, which cleaves pro-IL-1*β* to release IL-1*β* [29]. We primed macrophages with lipopolysaccharide (LPS) and activated inflammasomes with FAC, which resulted in the secretion of IL-1*β* as well as ROS (Figures 4(e) and 4(f)). Coincubation with ferrostatin, rotenone, and NAC abrogated FAC-mediated IL-1*β* and ROS production (Figures 4(e) and 4(f)). Altogether, we confirmed that FAC-mediated OXPHOS and the Fenton reaction are critical factors enhancing PD-L1 and IL-1*β* production.

## 4. Supplementary Description

### 4.1. FAC Enhances PD-L1 Expression during Macrophage Polarization

We examined the role of FAC in PD-L1 expression during polarization and confirmed that PD-L1 expression increased in both the M1 and M2 polarizing conditions (Supplementary Figure 1A–1D). Interestingly, PD-L1 expression was upregulated even in the CD86 downregulating condition (Supplementary Figure 1A and 1B).

### 4.2. FAC Enhances the Expression of PD-L1 in Various Cells

We next questioned if it could be applied to other cells as well as macrophages. We confirmed that the cancer cell line, CD11c^+^ dendritic cell, and CD3^+^ T cell also exhibited enhanced ROS production and upregulated PD-L1 following overnight incubation of 5 mM FAC (Supplementary Figure 2).

## 5. Discussion

Iron-induced metabolic and redox status renders an immunosuppressive macrophage phenotype. In this study, we observed that iron enhances PD-L1 expression in macrophages. We also pointed out that FAC may simultaneously manifest both the M1- and M2-like features and suggested that iron-mediated ROS production may lead to seemingly contradictory phenomena. The effect of FAC on ROS production and PD-L1 expression was not limited to macrophages. The inhibition of lipid peroxidation and ETC by a complex I inhibitor significantly abrogated FAC-mediated IL-1*β* production and PD-L1 expression (Figure 4). These findings show that the iron regulation of mitochondrial function, as iron increases lipid peroxidation and OXPHOS in mitochondria, is directly related to the increase of PD-L1 expression. Our findings do not necessarily mean that the metabolic alteration of FAC is the sole factor that determines the inflammatory pathway of macrophages, but rather, they highlight the need to consider metabolic and redox features when explaining the immunologic signals within macrophages. Thus, we confirmed that FAC-mediated OXPHOS and the Fenton reaction are critical factors for enhancing PD-L1 expression and IL-1*β* production.

Metabolically, M1 macrophages undergo a metabolic shift from aerobic glycolysis to OXPHOS, whereas the anti-inflammatory M2 phenotype requires maintenance of the OXPHOS pathway [2–5]. PD-L1 is a well-described immunosuppressive costimulatory molecule, which activates the immunoinhibitory PD-1/PD-L1 checkpoint, leading to impaired T cell immunity [30]. Recently, several studies have focused on the “mitochondrial OXPHOS-ROS-PD-L1” sequence in macrophages and T cells [11, 12, 31]. Scharping et al. successfully demonstrated that PD-1 expression in CD8^+^ T cells was derived from the maintenance of OXHOS [31]. We provided additional evidence that PD-L1 expression in macrophages is also dependent on OXPHOS by using rotenone (Figure 4(d)).

Patients with cancer often develop iron deficiency as a result of their underlying disease [32]. As an iron-deficient state or anemia has a negative impact on the cardiovascular system, physicians often correct the iron status by using iron supplements [33]. However, little is known about the effects of iron supplements on anticancer interventions, including anti-PD-L1 therapy in cancer patients [34]. Tymoszuk et al. recently demonstrated that iron overload directly inhibited CD8^+^ T cell activation and that the therapeutic effect of the anti-PD-L1 antibody was diminished in C57BL/6J female mice that were implanted with E0771 mammary carcinoma [34]. In addition to the direct impact of iron on CD8^+^ T cells, we suggested that iron mediates the upregulation of PD-L1 in antigen-presenting cells, which in turn suppresses CD4^+^ or CD8^+^ T cell activation and proliferation (Figure 1). Moreover, we demonstrated the impact of iron-overloaded macrophages on the regulatory phenotype in CD4^+^ T cells (Figure 2). Altogether, we have shown the effect of iron on PD-L1 upregulation in macrophages and have suggested ROS formation as an underlying mechanism. The findings of our study imply that iron supplementation in cancer patients with anemia may alter the therapeutic effect of anti-PD-L1 therapy. We provide evidence that iron supplementation contributes to the upregulation of PD-L1 expression in antigen-presenting cells, cancer cells, etc. Although we examined the impact of iron-mediated PD-L1 upregulation in macrophages on T cell activation, which in turn hampers anticancer therapy, other factors may determine the efficacy of anti-PD-L1 therapy. For example, the therapeutic effect of the anti-PD-L1 antibody increases as the PD-L1 expression level increases in cancer cells [35]. Thus, further clinical studies are needed to investigate the consequences of concurrent iron supplementation during anti-PD-L1 therapy in cancer patients.

## 6. Conclusions

In this study, we showed that ferric ammonium citrate (FAC) induces PD-L1 by modulating the metabolic and redox status of macrophages. FAC enhanced PD-L1 expression while also functionally impacting the regulatory phenotype on CD4^+^ T cells. FAC-overloaded macrophages showed an increased oxygen consumption rate (OCR), which was supported by the enhancement of aconitase activity and facilitation of the Fenton reaction. Using the Fenton reaction inhibitor and the complex I inhibitor, we confirmed that metabolic and redox regulation is responsible for FAC-mediated PD-L1 expression. In conclusion, the findings of our study, together with the reported results of previous investigations, are clinically meaningful in providing an insight into mechanisms that can potentially enhance the therapeutic effect of immune checkpoint inhibitors through the modulation of iron status in cancer patients.

## Data Availability

The data used to support the findings of this study are included within the article.

## Supplementary Materials

Supplementary Materials Supplementary Figure 1: effects of FAC on the expression of PD-L1 during macrophage polarization. BMDMs were polarized toward either M1 (mixture of 100 ng/mL LPS and 10 ng/mL IFN*γ*) or M2 (20 ng/mL IL4) and concurrently incubated with 5 mM FAC for 16 h. M1 and M2 polarization was confirmed by CD86 and CD206, respectively. PD-L1 expression in F4/80^+^ macrophages was measured by flow cytometry in M1 (A, B) and M2 (C, D). The contour line within the bar graph illustrates the mean fluorescence intensity (MFI) of each surface marker. ∗ indicates significant differences (^∗^*P* < 0.05, ^∗∗^*P* < 0.01, and ^∗∗∗^*P* < 0.001) in comparison with the control group. Supplementary Figure 2: effects of FAC on the expression of the PD-L1 in various cells. 1 × 10^5^ B16F1 cell line and splenocytes were treated with 5 mM FAC for 16 h. The PD-L1 expression and ROS production in the B16F1 cell line (A and B), CD11c^+^ dendritic cells (DC), and CD3^+^ T cells were compared with those in the control group. ∗ indicates significant differences (^∗^*P* < 0.05, ^∗∗^*P* < 0.01, and ^∗∗∗^*P* < 0.001) in comparison with the control group.

## References

[1] Wynn T. A., Chawla A., Pollard J. W. (2013). Macrophage biology in development, homeostasis and disease. *Nature*.

[2] Buck M. D., O'Sullivan D., Pearce E. L. (2015). T cell metabolism drives immunity. *The Journal of Experimental Medicine*.

[3] Buck M. D., Sowell R. T., Kaech S. M., Pearce E. L. (2017). Metabolic instruction of immunity. *Cell*.

[4] Choi E. J., Jeon C. H., Park D. H., Kwon T. H. (2020). Allithiamine exerts therapeutic effects on sepsis by modulating metabolic flux during dendritic cell activation. *Molecules and Cells*.

[5] Van den Bossche J., O'Neill L. A., Menon D. (2017). Macrophage immunometabolism: where are we (going)?. *Trends in Immunology*.

[6] Williams N. C., O'Neill L. A. J. (2018). A role for the Krebs cycle intermediate citrate in metabolic reprogramming in innate immunity and inflammation. *Frontiers in Immunology*.

[7] Emptage M. H., Kent T. A., Kennedy M. C., Beinert H., Munck E. (1983). Mossbauer and EPR studies of activated aconitase: development of a localized valence state at a subsite of the [4Fe-4S] cluster on binding of citrate. *Proceedings of the National Academy of Sciences of the United States of America*.

[8] Juang H. H. (2004). Modulation of iron on mitochondrial aconitase expression in human prostatic carcinoma cells. *Molecular and Cellular Biochemistry*.

[9] Infantino V., Iacobazzi V., Menga A., Avantaggiati M. L., Palmieri F. (2014). A key role of the mitochondrial citrate carrier (SLC25A1) in TNF*α*\- and IFN*γ*-triggered inflammation. *Biochimica et Biophysica Acta*.

[10] Galvan-Pena S., O'Neill L. A. (2014). Metabolic reprograming in macrophage polarization. *Frontiers in Immunology*.

[11] Du L., Lin L., Li Q. (2019). IGF-2 preprograms maturing macrophages to acquire oxidative phosphorylation- dependent anti-inflammatory properties. *Cell Metabolism*.

[12] Watanabe R., Shirai T., Namkoong H. (2017). Pyruvate controls the checkpoint inhibitor PD-L1 and suppresses T cell immunity. *The Journal of Clinical Investigation*.

[13] Wei Y., Liang M., Xiong L., Su N., Gao X., Jiang Z. (2021). PD-L1 induces macrophage polarization toward the M2 phenotype via Erk/Akt/mTOR. *Experimental Cell Research*.

[14] Hoeft K., Bloch D. B., Graw J. A., Malhotra R., Ichinose F., Bagchi A. (2017). Iron loading exaggerates the inflammatory response to the toll-like receptor 4 ligand lipopolysaccharide by altering mitochondrial homeostasis. *Anesthesiology*.

[15] Palmieri E. M., Gonzalez-Cotto M., Baseler W. A. (2020). Nitric oxide orchestrates metabolic rewiring in M1 macrophages by targeting aconitase 2 and pyruvate dehydrogenase. *Nature Communications*.

[16] Sukhbaatar N., Weichhart T. (2018). Iron regulation: macrophages in control. *Pharmaceuticals*.

[17] Behmoaras J. (2021). The versatile biochemistry of iron in macrophage effector functions. *The FEBS Journal*.

[18] Kowaltowski A. J., de Souza-Pinto N. C., Castilho R. F., Vercesi A. E. (2009). Mitochondria and reactive oxygen species. *Free Radical Biology & Medicine*.

[19] Ni S., Kuang Y., Yuan Y., Yu B. (2020). Mitochondrion-mediated iron accumulation promotes carcinogenesis and Warburg effect through reactive oxygen species in osteosarcoma. *Cancer Cell International*.

[20] Zhao R. Z., Jiang S., Zhang L., Yu Z. B. (2019). Mitochondrial electron transport chain, ROS generation and uncoupling (review). *International Journal of Molecular Medicine*.

[21] Latunde-Dada G. O. (2017). Ferroptosis: role of lipid peroxidation, iron and ferritinophagy. *Biochimica et Biophysica Acta - General Subjects*.

[22] Agoro R., Taleb M., Quesniaux V. F. J., Mura C. (2018). Cell iron status influences macrophage polarization. *PLoS One*.

[23] Handa P., Thomas S., Morgan-Stevenson V. (2019). Iron alters macrophage polarization status and leads to steatohepatitis and fibrogenesis. *Journal of Leukocyte Biology*.

[24] Nakamura K., Kawakami T., Yamamoto N. (2016). Activation of the NLRP3 inflammasome by cellular labile iron. *Experimental Hematology*.

[25] Wang H., Li Z., Niu J. (2018). Antiviral effects of ferric ammonium citrate. *Cell Discovery*.

[26] Schmidt A., Zhang X. M., Joshi R. N. (2016). Human macrophages induce CD4+Foxp3+ regulatory T cells via binding and re- release of TGF-*β*. *Immunology and Cell Biology*.

[27] Rossi G. A., Zocchi E., Sacco O., Balbi B., Ravazzoni C., Damiani G. (1986). Alveolar macrophage stimulation of T-cell proliferation in autologous mixed lymphocyte reactions. *The American Review of Respiratory Disease*.

[28] Vogt A. S., Arsiwala T., Mohsen M., Vogel M., Manolova V., Bachmann M. F. (2021). On iron metabolism and its regulation. *International Journal of Molecular Sciences*.

[29] Kelley N., Jeltema D., Duan Y., He Y. (2019). The NLRP3 inflammasome: an overview of mechanisms of activation and regulation. *International Journal of Molecular Sciences*.

[30] Francisco L. M., Salinas V. H., Brown K. E. (2009). PD-L1 regulates the development, maintenance, and function of induced regulatory T cells. *The Journal of Experimental Medicine*.

[31] Scharping N. E., Rivadeneira D. B., Menk A. V. (2021). Mitochondrial stress induced by continuous stimulation under hypoxia rapidly drives T cell exhaustion. *Nature Immunology*.

[32] Maccio A., Madeddu C., Gramignano G. (2015). The role of inflammation, iron, and nutritional status in cancer-related anemia: results of a large, prospective, observational study. *Haematologica*.

[33] Abdel-Razeq H., Hashem H. (2020). Recent update in the pathogenesis and treatment of chemotherapy and cancer induced anemia. *Critical Reviews in Oncology/Hematology*.

[34] Tymoszuk P., Nairz M., Brigo N. (2020). Iron supplementation interferes with immune therapy of murine mammary carcinoma by inhibiting anti-tumor T cell function. *Frontiers in Oncology*.

[35] Xu Y., Wan B., Chen X. (2019). The association of PD-L1 expression with the efficacy of anti-PD-1/PD-L1 immunotherapy and survival of non-small cell lung cancer patients: a meta-analysis of randomized controlled trials. *Translational Lung Cancer Research*.

