# Supplementary material for: Ferric Ammonium Citrate Upregulates PD-L1 Expression through Generation of Reactive Oxygen Species
Source: J Immunol Res. 2022 Jan 17;2022:6284124. doi: 10.1155/2022/6284124 (PMC8786474; doi:10.1155/2022/6284124)
Supplement: Supplementary Materials — Supplementary Figure 1: effects of FAC on the expression of PD-L1 during macrophage polarization. BMDMs were polarized toward either M1 (mixture of 100 ng/mL LPS and 10 ng/mL IFNγ) or M2 (20 ng/mL IL4) and concurrently incubated with 5 mM FAC for 16 h. M1 and M2 polarization was confirmed by CD86 and CD206, respectively. PD-L1 expression in F4/80+ macrophages was measured by flow cytometry in M1 (A, B) and M2 (C, D). The contour line within the bar graph illustrates the mean fluorescence intensity (MFI) of each surface marker. ∗ indicates significant differences (∗P < 0.05, ∗∗P < 0.01, and ∗∗∗P < 0.001) in comparison with the control group. Supplementary Figure 2: effects of FAC on the expression of the PD-L1 in various cells. 1 × 10^5^ B16F1 cell line and splenocytes were treated with 5 mM FAC for 16 h. The PD-L1 expression and ROS production in the B16F1 cell line (A and B), CD11c+ dendritic cells (DC), and CD3+ T cells were compared with those in the control group. ∗ indicates significant differences (∗P < 0.05, ∗∗P < 0.01, and ∗∗∗P < 0.001) in comparison with the control group. [file 6284124.f1.docx]

Supplementary Fig. 1: Effects of FAC on the expression of PD-L1 during macrophage polarization. BMDMs were polarized toward either M1 (mixture of 100ng/mL LPS and 10ng/mL IFNγ) or M2 (20ng/mL IL4), and concurrently incubated with 5 mM FAC for 16 h. M1 and M2 polarization was confirmed by CD86 and CD206, respectively. PD-L1 expression in F4/80^+^macrophages was measured by flow cytometry in M1 (A, B) and M2 (C, D). The contour line within the bar graph illustrates the mean fluorescence intensity (MFI) of each surface marker. * indicates significant differences (* P < 0.05, ** P < 0.01, *** P < 0.01) in comparison with the control group.

Supplementary Fig. 2: Effects of FAC on the expression of PD-L1 in various cells. 1x10^5^ B16F1 cell line and splenocytes were treated with 5 mM FAC for 16 h. The PD-L1 expression and ROS production in the B16F1 cell line (A and B), CD11c^+^ dendritic cells (DC), and CD3^+^ T cells were compared with that in the control group. * indicates significant differences (* P < 0.05, ** P < 0.01, *** P < 0.01) in comparison with the control group.
